# Small Vessel Disease and Dietary Salt Intake: Cross-Sectional Study and Systematic Review

**DOI:** 10.1016/j.jstrokecerebrovasdis.2017.08.004

**Published:** 2017-12

**Authors:** Stephen D.J. Makin, Ghaida F. Mubki, Fergus N. Doubal, Kirsten Shuler, Julie Staals, Martin S. Dennis, Joanna M. Wardlaw

**Affiliations:** * Centre for Clinical Brain Sciences, Edinburgh, United Kingdom; † Academic Section of Geriatric Medicine, Institute of Cardiovascular and Medical Sciences, University of Glasgow, Glasgow, United Kingdom; ‡ Edinburgh Dementia Research Centre in the UK Dementia Research Initiative, Edinburgh, United Kingdom; § Department of Neurology, Maastricht University Medical Center, Maastricht, The Netherlands

**Keywords:** Dietary salt, urinary sodium/creatinine ratio, white matter lesions, acute stroke

## Abstract

**Background:**

Higher dietary salt intake increases the risk of stroke and may increase white matter hyperintensity (WMH) volume. We hypothesized that a long-term higher salt intake may be associated with other features of small vessel disease (SVD).

**Methods:**

We recruited consecutive patients with mild stroke presenting to the Lothian regional stroke service. We performed brain magnetic resonance imaging, obtained a basic dietary salt history, and measured the urinary sodium/creatinine ratio. We also carried out a systematic review to put the study in the context of other studies in the field.

**Results:**

We recruited 250 patients, 112 with lacunar stroke and 138 with cortical stroke, with a median age of 67.5 years. After adjustment for risk factors, including age and hypertension, patients who had not reduced their salt intake in the long term were more likely to have lacunar stroke (odds ratio [OR], 1.90; 95% confidence interval [CI], 1.10-3.29), lacune(s) (OR, 2.06; 95% CI, 1.09-3.99), microbleed(s) (OR, 3.4; 95% CI, 1.54, 8.21), severe WMHs (OR, 2.45; 95% CI 1.34-4.57), and worse SVD scores (OR, 2.17; 95% CI, 1.22-3.9). There was limited association between SVD and current salt intake or urinary sodium/creatinine ratio. Our systematic review found no previously published studies of dietary salt and SVD.

**Conclusion:**

The association between dietary salt and background SVD is a promising indication of a potential neglected contributory factor for SVD. These results should be replicated in larger, long-term studies using the recognized gold-standard measures of dietary sodium.

## Introduction

Cerebral small vessel disease (SVD) is common and clinically important: it is responsible for dementia,^1^ depression,^2^ physical disability,^3^ and one fifth of all strokes.^4^ On pathology, there is an intrinsic disease of the small deep-penetrating arteries with appearances described as segmental arteriolar disorganization, fibrinoid necrosis, and lipohylinosis.^5^ However, the etiology is unclear: while common vascular risk factors such as hypertension are very important, they only account for a small proportion of the variance in SVD features.^6^

Previously, we found that increased dietary salt intake was associated with greater white matter hyperintensity (WMH) volumes, a key feature of SVD.^7^ Dietary salt also increases the risk of any type of stroke: in 19 cohort studies (n > 170,000 participants), the relative risk of stroke was 1.23 (95% confidence interval [CI], 1.06-1.4) for higher versus lower salt intake,^8^ confirmed in the North Manhattan Stroke Study, which found a 17% increase in the 10-year stroke risk for each additional 500 mg of daily dietary sodium.^9^

To further examine the potential relationship between dietary salt intake and SVD clinical and imaging features, we performed a cross-sectional study in patients presenting with minor stroke and a systematic review of the existing literature.

## Materials and Methods

We recruited consecutive inpatients and outpatients presenting to our regional stroke service with a minor ischemic stroke from May 2010 to May 2012. We defined “minor” as a stroke with a National Institutes of Health Stroke Scale (NIHSS) score of 7 or lower at assessment and which was not anticipated to cause impairment in activities of daily living. The detailed recruitment and data collection procedure have been published previously.^7^, ^10^

The study was approved by the Lothian Ethics of Medical Research Committee (REC 09/81,101/54) and National Health Service Lothian Research and Development Office (2009/W/NEU/14) according to the Health Research Authority, United Kingdom, which uses the Declaration of Helsinki 1975 (revised in 1983), and all patients gave written informed consent.

All patients underwent magnetic resonance imaging (MRI) as soon as possible after presentation (1.5 Tesla GE Scanner, General Electric Medical Systems, Milwaukee, USA) with T1, T2, T2*, fluid-attenuated inversion recovery, and diffusion-weighted imaging sequences.

An experienced neuroradiologist (J.M.W.) reviewed all imaging and classed the stroke lesion as “lacunar” or “cortical” blind to clinical features; if no lesion was present, the stroke syndrome was classified on clinical findings based on the Bamford Classification.^7^ We defined hypertension as either a previous diagnosis of hypertension made prior to the stroke or a diagnosis of hypertension made after presentation with stroke. All patients had blood pressure (BP) recordings made during their NHS clinical care. We recorded deep and periventricular WMHs on the Fazekas Scale, and a total SVD burden score (0-4) that incorporates microbleeds, lacunes, periventricular spaces in the basal ganglia, and WMH, as described previously,^11^ classifying severe WMHs as a periventricular Fazekas score of 3 and/or a deep Fazekas score of 2-3.

We assessed dietary salt intake during patient recruitment with the following questions. The first 2 questions were adapted from a widely used validated food frequency questionnaire,^12^ and the third question we derived ourselves as there was no widely used validated question to estimate if dietary salt intake had reduced during adult life:

a. *“Do you/your partner add salt to your food during cooking”: Always/Often/Occasionally/Rarely/Never*
b. *“Do you add salt to your food at the table”: Always/Often/Occasionally/Rarely/Never*
c. *“Have you reduced the amount of salt that you add to your food, during cooking or at the table, since age 20”: No take more salt/Yes, I take less salt/I take the same amount of salt.*

These questions were chosen pragmatically to identify patients who were likely to have a higher salt intake from those who on average were likely to have a lower salt intake, rather than to quantify lifetime salt intake. The gold-standard measurement of current dietary salt with a detailed food frequency questionnaire or 24-hour urine would have been impractical in the context of a large study of patients with a recent stroke.

We measured the urinary sodium : creatinine ratio on an early-morning urine specimen obtained a minimum of 1 month post–index stroke using the standard hospital biochemistry laboratory procedures.

We performed all statistical analyses using R. We used Fisher's exact test to compare dichotomous variables, the Wilcox test, the Mann–Whitney test for nonparametric continuous variables, Student's *t*-test, and Spearman's Rho for parametric tests. As this was an exploratory secondary analysis, no power calculation was performed. The answers to questions *a* and *b* were scored as 0-4 with 0 being “never adds salt” and 4 being “always adds salt”; the score for both questions was added together to make a “Total Current Salt Score” of 0-8. We summed the Fazekas deep (0-3) and periventricular (0-3) WMH scores into a “Total Fazekas Score” (0-6). We performed a multivariable logistic regression analysis, the choice of variables being informed by known associations and univariable analysis results.

### Systematic Review

We searched MEDLINE, EMBASE (from 1947 to present), LILACS, CINAHL, and psychINFO using search terms for dietary salt and SVD, and hand-searched conference abstracts and review article references (search terms in the Supplement). We contacted authors of those studies who had measured salt and stroke to ask if they had unpublished data on stroke subtypes or imaging variables.

## Results

**Figure 1:**
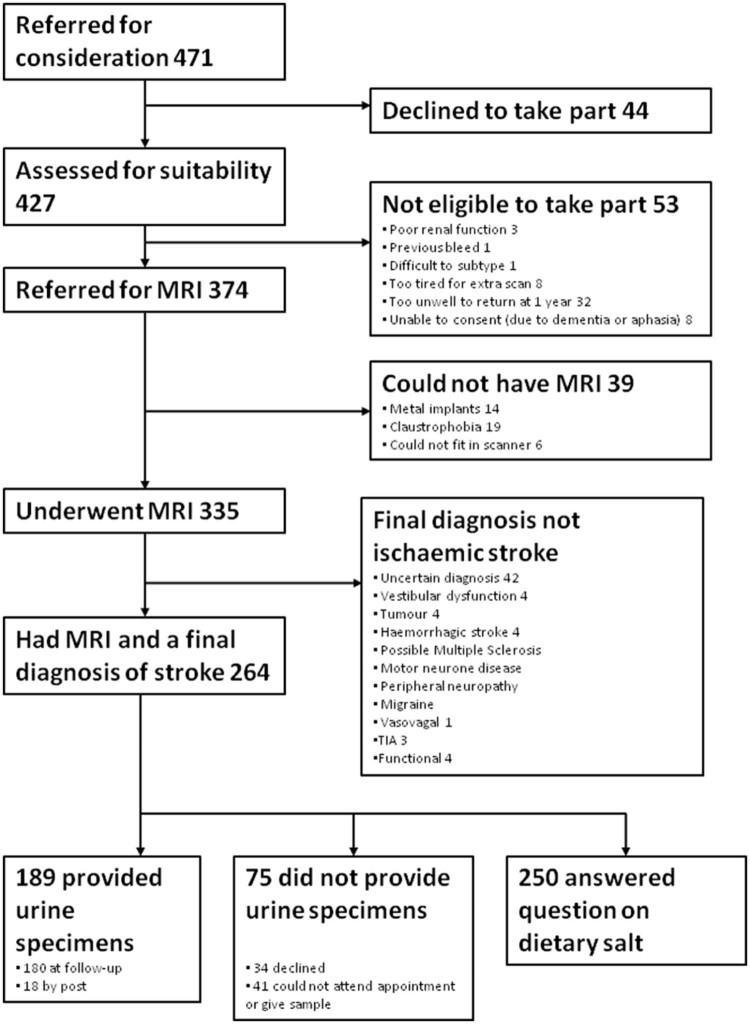
Patient recruitment. Abbreviation: MRI, magnetic resonance imaging; TIA, transient ischemic attack.

Of the 471 patients referred for the study, 264 were included (44 declined, 39 could not have MRI, 71 had a final diagnosis other than stroke, and 53 were not eligible for other reasons; Fig 1), 2 did not answer the question about current salt, and 14 did not answer if they had reduced salt. Therefore, data from 250 patients were included in this analysis. The median age of patients was 67.5, 37% were female and the median NIHSS score was 2 (interquartile range, 2-3) (Table 1). There were 189 patients who provided a urine specimen; men, younger patients, and those with lower NIHSS scores were more likely to provide a urine sample (Table S1).

**Table 1:** Characteristics of patients, and univariable analysis

	All patients	Had not cut down on salt over lifetime n = 146	Had cut down on salt over lifetime n = 104	*P* (unadjusted difference in salt reduction
Demographic features				
Female gender	73 (37%)	60 (41%)	44 (42%)	.9
Median age at index stroke	67.5 (IQR 60, 75.25)	66 (IQR 57-72)	66 (IQR 56-75)	.6
Dietary salt				
Added salt at the table (1-5)*	1 (0,2)	1 (0-3)	0 (0-2)	***<.001***
Added salt during cooking (1-5)*	2 (0,4)	2 (1-4)	1 (0-3)	***<.001***
Median total salt score (IQR)	4 (1-5)	4 (2-6)	2 (0-4)	***<.001***
Stroke-related features				
Lacunar subtype	90 (46%)	75 (51%)	37 (36%)	***.01***
Median NIHSS at worse point (IQR)	2 (1,3)	2 (1-3)	2 (2-3)	.9
Stroke risk factors				
AF	23 (9%)	9 (6%)	14 (13%)	.07
Carotid stenosis on either side	26 (10%)	10 (8%)	16 (17%)	1
Smoker or stopped within last 12 months	96 (36%)	65 (45%)	32 (31%)	***.03***
Past medical history				
Known diabetes	27 (14%)	13 (9%)	15 (14%)	.22
Diagnosis of hypertension prior to index stroke (or diagnosed at presentation)	142 (72%)	98 (67%)	83 (80%)	.03
Median systolic BP at presentation†	150 (27.9)	151 (29)	151 (25)	.92
Median diastolic BP at presentation†	83.7 (14.4)	84.4 (14.3)	82 (14.0)	.44
Diagnosis of hyperlipidemia prior to index stroke (or diagnosed at presentation)	85 (43%)	81 (55%)	71 (68%)	***.05***
Ischaemic heart disease	41 (21%)	24 (16%)	25 (24%)	.15
Peripheral vascular disease	14 (5%)	8 (5%)	6 (6%)	1
Previous TIA prior to index stroke	28 (11%)	16 (11%)	12 (12%)	1
Previous stroke prior to index stroke	30 (11%)	17 (12%)	13 (13%)	.85
Any large vessel disease (IHD, PVD, or carotid stenosis on either side)	75 (28%)	39 (27%)	36 (35%)	.21
Imaging features : SVD score				
Median total SVD score	1 (0-2)	1 (0-2)	1 (0-2)	.01
At least 1 microbleed	42 (16%)	33 (23%)	9 (9%)	***.003***
PVS in the basal ganglia	123 (47%)	74 (51%)	49 (47%)	.61
Periventricular WMH score 1+	110 (42%)	73 (50%)	37 (36%)	***.03***
At least 1 lacunae on index scan	66 (25%)	46 (32%)	20 (19%)	***.04***
Imaging features: median Fazekas Score				
Median periventricular score (IQR)	1 (1,2)	1 (1-2)	1 (1-2)	.25
Median deep score (IQR)	1 (1,2)	1 (1-2)	1 (1-2)	.45
Median total score (IQR)	3 (2-5)	3 (2-4)	2 (2-4)	.37

Abbreviations: AF, atrial fibrillation; BP, blood pressure; IHD, ischaemic heart disease; IQR, interquartile range; NIHSS, National Institutes of Health Stroke Scale; PVD, peripheral vascular disease; PVS, perivascular spaces; SVD, small vessel disease; TIA, transient ischemic attack; WMH, white matter hyperintensity.

Bold and Italic indicate statistical significance.

* Median (IQR).

† mm Hg (IQR).

The 104 patients who indicated that they had reduced their salt intake in adult life reported adding less salt to food currently than the 146 patients who had not cut back (Table 1).

Patients who had reduced dietary salt during adulthood were more likely to have a diagnosis of hypertension (80% versus 67% who had not reduced salt, *P* = .03) or hyperlipidemia (71% versus 81% of those who had not reduced salt, *P* = .05) and were also less likely to smoke (31% versus 45%, *P* = .03).

Patients who had *not* reduced salt during adult life were more likely to have the lacunar stroke subtype (51% versus 36%, *P* = .01), severe WMHs (50% versus 36%, *P* = .03), microbleeds (23% versus 9%, *P* = .003), and a higher total SVD score.

On multivariable analysis (Table 2), there was still a significant association between having *not* reduced salt and lacunar stroke (odds ratio [OR], 1.90; 95% CI 1.10-3.29), lacunes (OR, 2.06; 95% CI, 1.09-3.99), severe WMHs (OR, 2.45; 95% CI, 1.34-4.57), microbleeds (OR, 3.4; 95% CI, 1.54, 8.21), and high SVD scores (2 or more; OR, 2.17; 95% CI, 1.22-3.9). There was no significant relationship between salt intake and periventricular spaces on univariate or multivariate analysis.

**Table 2:** Multivariable analysis of the relationship between having not cut back on salt and different imaging features of small vessel disease

	Model 1 unadjusted	Model 2 adjusted for age and sex	Model 3, adjusted for age, sex, and vascular risk factors
Odds ratio of lacunar stroke (versus nonlacunar stroke)			
Had not cut back on salt	***1.91 (95% CI, 1.15-3.22)***	***1.88 (95% CI, 1.12-3.19)***	***1.90 (95% CI, 1.10-3.29)***
Age		***.97 (95% CI, .94-.99)***	.97 (95% CI, .95-1.00)
Sex (male)		.88 (95% CI, .52-1.50)	.94 (95% CI, .55-1.60)
Hypertension			1.36 (95% CI, .74-2.53)
Smoking last year			1.27 (95% CI, .71-2.26
AF			.56 (95% CI, .17-1.48)
PVD			.21 (95% CI, .31-.81)
IHD			.90 (95% CI, .44-1.81)
Hyperlipidemia			1.21 (95% CI, .70-2.11)
Odds ratio of having a lacunae on the index scan			
Had not cut back on salt	***1.93 (95% CI, 1.07-3.57)***	***1.94 (95% CI, 1.07, 3.62)***	***2.06 (95% CI, 1.09-3.99)***
Age		.99 (95% CI, .97, 1.01)	1 (95% CI, .97-1.02)
Male gender		.95 (95% CI, .56, 1.6)	2.28 (95% CI, 1.21-4.42)
Known hypertension			.57 (95% CI, .3, 1.07)
Smoker (or recent ex-smoker)			1.56 (95% CI, .87, 2.81)
AF			.6 (95% CI, .16-1.81)
PVD			2.13 (95% CI, .62-7.05)
IHD			1.4 (95% CI, .64-2.96)
Hyperlipidemia			.61 (95% CI, .34, 1.06)
Odds ratio of WMH score 1 on SVD score			
Had not cut back on salt	***1.81 (95% CI, 1.08, 3.05)***	***2.29 (95% CI, 1.29-4.14)***	***2.45 (95% CI, 1.34-4.57)***
Age		***1.09 (95% CI, 1.06-1.13)***	***1.09 (95% CI, 1.06-1.13)***
Male gender		.88 (95% CI, .5-1.56)	.82 (95% CI, .45-1.47)
Known hypertension			2.49 (95% CI, 1.25-5.15)
Smoker (or recent ex-smoker)			1.45 (95% CI, .77-2.79)
AF			.93 (95% CI, .35-2.54)
PVD			2.1 (95% CI, .63-8.37)
IHD			1.21 (95% CI, .58-2.53)
Hyperlipidemia			.87 (95% CI, .47-1.61)
Odds ratio of having EPVL in the basal ganglia			
Had not cut back on salt	1.15 (95% CI, .7, 1.91)	1.27 (95% CI, .74-2.2)	1.22 (95% CI, .69-2.16)
Age		1.07 (95% CI, 1.05-1.1)	1.08 (95% CI, 1.05-1.11)
Male gender		1.68 (95% CI, .97-2.92)	1.62 (95% CI, .93-2.85)
Known hypertension			1.39 (95% CI, .73-2.66)
Smoker (or recent ex-smoker)			1.62 (95% CI, .88-3.02)
AF			.95 (95% CI, .37-2.56)
PVD			.78 (95% CI, .24-2.6)
IHD			1.07 (95% CI, .53-2.19)
Hyperlipidemia			.94 (95% CI, .53-1.66)
Odds ratio of having, at least, one microbleed			
Had not cut back on salt	***3.08 (95% CI, 1.46, 7.14)***	***3.35 (95% CI, 1.56, 7.89)***	***3.4 (95% CI, 1.54, 8.21)***
Age		.99 (95% CI, .96, 1.01)	1 (95% CI, .98, 1.03)
Male gender		.98 (95% CI, .58, 1.65)	.98 (95% CI, .56, 1.69)
Known hypertension			.55 (95% CI, .29, 1.03)
Smoker (or recent ex-smoker)			1.62 (95% CI, .9, 2.95)
AF			.58 (95% CI, .22, 1.47)
PVD			.9 (95% CI, .27, 3.06)
IHD			.83 (95% CI, .41, 1.66)
Hyperlipidemia			.59 (95% CI, .33, 1.03)
Odds ratio of having an SVD score of 2 or more			
Had not cut back on salt	***1.84 (95% CI, 1.1-3.1)***	***2.07 (95% CI, 1.2-3.6)***	***2.17 (95% CI, 1.22-3.9)***
Age		.98 (95% CI, .96-1.01)	1 (95% CI, .97-1.03)
Male gender		.97 (95% CI, .58-1.64)	1 (95% CI, .58-1.73)
Known hypertension			.55 (95% CI, .28-1.02)
Smoker (or recent ex-smoker)			1.52 (95% CI, .85-2.76)
AF			.52 (95% CI, .2-1.31)
PVD			.87 (95% CI, .27-2.92)
IHD			.87 (95% CI, .27-2.92)
Hyperlipidemia			.64 (95% CI, .36-1.11)

Abbreviations: AF, atrial fibrillation; CI, confidence interval; IHD, ischaemic heart disease; PVD, peripheral vascular disease; SVD, small vessel disease.

***Bold italics*** indicate *P* < .05.

A higher total current salt score was associated with a slightly higher total Fazekas^13^ score on univariable analysis, but this became nonsignificant when adjusted for age. There was no other association between total current salt score and SVD features (Table S3).

**Figure 2:**
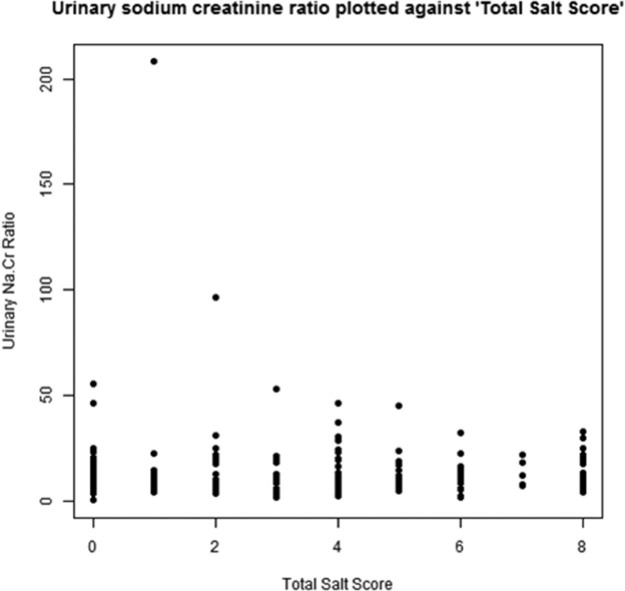
Urinary sodium : creatinine ratio plotted against “Total Current Salt Score.”

The urinary sodium : creatinine ratio was associated with age, diuretic use, diagnosis of hypertension, and reduced eGFR. Urinary sodium correlated poorly with the reported current dietary salt, R = −.01, *P* = .94 (Fig 2), or with whether or not the patients had reduced their salt in adulthood. There was no association between the urinary sodium/creatinine ratio and SVD features (Tables S2, S3, and S4).

**Figure 3:**
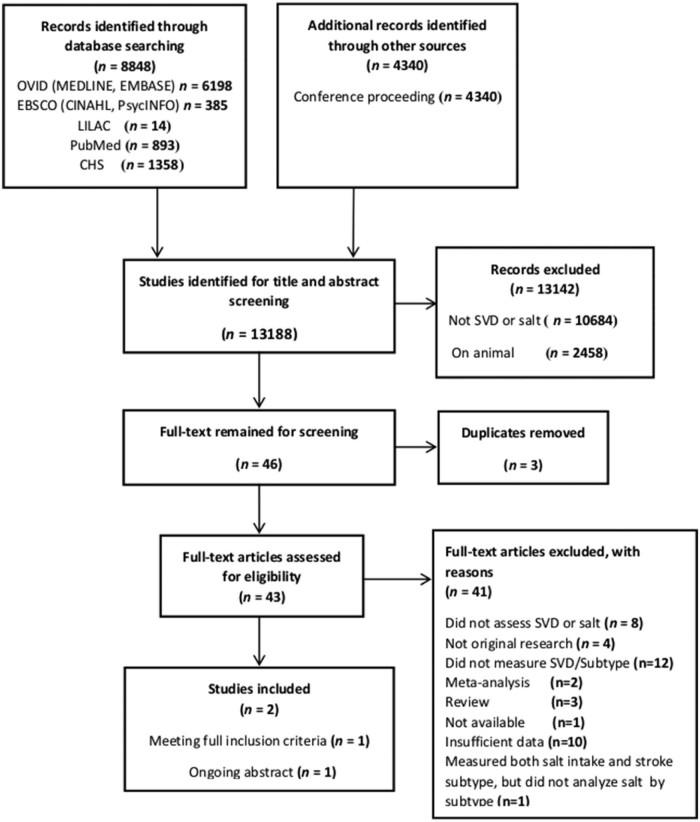
Papers identified for the systematic review.

The systematic literature search found 13,188 potential titles, of which 2458 were animal studies and 10,684 did not measure either dietary salt or SVD (Fig 3). We reviewed 46 full-text articles, of which 2 did not measure salt or SVD, 27 did not provide data on dietary sodium, and 12 provided data on only all-cause stroke. We contacted 3 authors of studies^9^, ^14^, ^15^ that had measured all-cause stroke and salt intake, but they did not have any data available regarding stroke subtyping. The only study we identified that provided data on salt and SVD in humans was the present study when published in a conference abstract and a separate analysis of WMH volume in the same patient group.^7^

## Discussion

Patients presenting with the lacunar ischemic stroke subtype were less likely to report that they had reduced their dietary salt during adult life than patients with cortical stroke. Not reducing salt during adulthood was also associated with a higher total SVD score and more often having lacunes, worse WMHs, or microbleeds. This association remained after adjustment, including for hypertension and age. There was little association between stroke subtype, or SVD features, and the urine sodium : creatinine ratio or a total current salt score. This is the first time, to our knowledge, that the relationship between dietary salt, SVD features (other than WMH volume), and the lacunar stroke subtype has been reported. It supports our previous finding that higher reported salt intake (the total current salt score) was associated with a larger WMH volume.^7^

There were limitations. We did not use a detailed dietary history to assess current salt intake because dietary salt is acknowledged to be difficult to assess, partly due to the amount of salt in processed food. It would have been impractical to perform complex detailed dietary measures or expect 24-hour urine collections in this group of patients with recent stroke. The measurement of dietary sodium in patients after stroke is challenging and difficult to do without bias,^16^ any method that involves asking patients details about their diet is prone to recall bias, and the use of urine measurements is likely to be confounded by diuretic medication with which many are prescribed.^17^ The gold-standard measurements of 24-hour urinary sodium, or a 5-day weighted food diary, are likely to be difficult to undertake for patients with stroke, even minor stroke, given other medical constraints.^18^ However, it is reasonable to suppose that patients who report having reduced salt have, on average, taken less salt over their lifetime than patients who have not reduced their intake, as having reduced salt intake was also related to a lower score on the question about current salt intake. Our dietary questions were intended to group patients into those with high and low salt intake rather than quantify dietary salt in an exact manner. We performed several analyses that may have increased the chance of detecting spurious associations with dietary salt. As such, the findings should be considered exploratory and require confirmation in another study.

One of the strengths of this study is that all patients were carefully assessed and thoroughly evaluated, with a diagnosis of stroke and stroke subtype made by a multidisciplinary panel and all MRI imaging reviewed by an experienced neuroradiologist. All such steps used validated protocols and were blinded to dietary and urinary salt data.

The association between having *not* cut down on salt and SVD may be due to patients who had *not* cut down on salt having a greater lifetime salt intake, or it may be due to confounding from other lifestyle changes that the patients may have adopted while cutting down on salt, such as increasing fruit and vegetable intake, stopping smoking, or increasing physical activity. Apart from smoking, which was included in the multivariable analysis, we were not able to assess other lifestyle factors. Additionally, patients who had reduced their salt intake were more likely to have a diagnosis of hypertension, which might be expected to increase the SVD burden, and such patients may have attempted to reduce their salt intake in response to standard dietary advice for hypertensive patients. The sample was not large enough to assess for factors such as time since hypertension diagnosis on the salt–SVD relationship. The lack of association with current urinary salt may represent confounding from the patients who took diuretic medication or a lack of statistical power to detect a statistically significant difference. The risk of false positives has been discussed. The findings should be assessed in larger studies that also assess lifestyle factors.

However, the results suggest that it would be interesting to assess prospectively the long-term effects of current dietary salt on future SVD burden while obtaining more detailed measures of current and previous salt intake. While it is notable that there was no relationship between current salt intake and the BP measured at presentation, a single measurement in a stressful situation such as hospital assessment may not be representative of underlying BP over many years.

The association between having *not* reduced dietary salt and SVD is consistent with other cerebrovascular risks of salt such as all-cause stroke. Future studies should assess for associations with specific stroke subtypes. Higher dietary salt intake has also been linked to a more rapid progression of multiple sclerosis. The effect of dietary salt on cerebral microvascular endothelial tight junctions and microglial activation in an experimental model are also worthy of further investigation.^19^

As with all novel findings, these cross-sectional analyses should be repeated in other data sets and assessed in larger prospective studies. The finding that stroke patients who have not reduced salt are more likely to have SVD is novel and worthy of further investigations.
