# Supplementary material for: Small Vessel Disease and Dietary Salt Intake: Cross-Sectional Study and Systematic Review
Source: J Stroke Cerebrovasc Dis. 2017 Dec;26(12):3020–8. doi: 10.1016/j.jstrokecerebrovasdis.2017.08.004 (PMC5711036; doi:10.1016/j.jstrokecerebrovasdis.2017.08.004)
Supplement: Appendix S1 — Tables S1-S4. [file mmc1.docx]

Supplementary information

Search Strategy

1. brain ischemia/ or brain infarction/ or brain stem infarctions/ or cerebral infarction/ or hypoxia-ischemia, brain/ or stroke/

2. (isch?emi$ adj6 (stroke$ or apoplex$ or cerebral vasc$ or cerebrovasc$ or cva or attack$)).tw.

3. ((brain or cerebr$ or cerebell$ or vertebrobasil$ or hemispher$ or intracran$ or intracerebral or infratentorial or supratentorial or middle cerebr$ or mca$ or anterior circulation) adj5 (isch?emi$ or infarct$ or thrombo$ or emboli$ or occlus$ or hypoxi$)).tw.

4. (lacun$ or small vessel$ or small infarct$ or microinfarct$ or subcortical lesion$ or subcortical infarct$ or microvascular$ or microcirculation$).tw.

5. 1, 2, 3, OR 4.

6. Sodium chloride, dietary

OR

(Salt adj diet). tw

5 AND 6

Table S1: patients who did and did not provide a urine sample.

|  | ***Specimen provided***  ***n=189***  ***(n/%)*** | ***No Specimen provided***  ***n=75  (n/%)*** | ***p*** |
| --- | --- | --- | --- |
| Female | 70(37%) | 40(53%) | 0.02 |
| Diabetes | 22(12%) | 8(11%) | 1 |
| Hypertension | 135(71%) | 52(69%) | 0.76 |
| Hyperlipidaemia | 119(63%) | 42(56%) | 0.33 |
| AF | 21(11%) | 4(5%) | 0.17 |
| IHD | 39(21%) | 14(19%) | 0.86 |
| Previous TIA | 17(9%) | 11(15%) | 0.19 |
| Previous Stroke | 23(12%) | 9(12%) | 1 |
| Lacunar | 86(46%) | 33(44%) | 1 |
| Age | 67(IQR 59,75) | 67(IQR 59,76) | 0.93 |
| NIHSS | 2(IQR 1,3) | 2(IQR 2,4) | 0.05 |

Table S2 Univariable analysis of patients’ total salt and urinary sodium

|  | | ***Total current salt score*** | | |  | |
| --- | --- | --- | --- | --- | --- | --- |
|  |  | ***All Subjects n=264*** | | |  |  |
|  | | ***Median(IQR)*** | ***Median (IQR)*** | | ***P*** | |
|  |  | ***with parameter*** | ***without parameter*** | |  |  |
| Female gender | | 4(IQR 2-5) F | 3(IQR 1-5) | | 0.185 | |
| Taking diuretics | | 4(IQR 1-5) | 4(IQR 1-5) | | 0.907 | |
| Lacunae on index scan | | 4(IQR 2-6) | 4(IQR 1-5) | | 0.432 | |
| Fazekas score for periventricular 2+ on index scan | | 4(IQR 2-6) | 3(IQR 1-4) | | 0.07 | |
| Moderate-severe EPVL in the basal ganglia | | 4(IQR 1-5) | 4(IQR 1-5) | | 0.691 | |
| At least one microbleed | | 4(IQR 1-6) | 4(IQR 1-5) | | 0.537 | |
| Lacunar subtype | | 4(IQR 2-6) | 3(IQR 1-5) | | 0.099 | |
| Hypertension | | 3(IQR 1-5) | 4(IQR 2-6) | | 0.069 | |
| Diabetes | | 3(IQR 0-4) | 4(IQR 1-5) | | 0.374 | |
| Stroke | | 4(IQR 2-6) | 4(IQR 1-5) | | 0.405 | |
| PVD | | 4(IQR 2-6) | 4(IQR 1-5) | | 0.296 | |
| eGFR <60 | | 2(IQR 1-4) | 4(IQR 1-5) | | 0.368 | |
| Had not cut down on salt | | 4(IQR 2-6) | 2(IQR 0-4) | | ***<0.001*** | |
|  | | ***Spearmans Rho*** | | ***P*** | | |
| Age | | 0.09 | | 0.157 | | |
| Systolic BP | | -0.031 | | 0.653 | | |
| Diastolic BP | | 0.004 | | 0.95 | | |
| NIHSS at worst point | | 0.11 | | 0.083 | | |
| Total Fazekas score | | 0.127 | | ***0.045*** | | |
| Total SVD score | | 0.083 | | 0.191 | | |
|  | | ***Measured Urinary Sodium Creatinine Ratio*** | | | | |
|  | | ***Subjects who provided a sample n=196*** | | | | |
|  | ***with parameter*** | | ***Without*** | | P |  |
| Female gender | 19.03(SD 27.13)f | | 12.09(SD 8.02) m | | ***0.014*** |  |
| Taking diuretics | 22.32(SD 32.64) | | 12.12(SD 7.46) | | **0.*018*** |  |
| Lacunae on index scan | 11.14(SD 7.53) | | 15.82(SD 20.13) | | ***0.018*** |  |
| Fazekas score for periventricular 2+ on index scan | 17.13(SD 24.22) | | 12.92(SD 11.49) | | 0.121 |  |
| Moderate-severe EPVL in the basal ganglia | 15.88(SD 22.18) | | 13.4(SD 12.12) | | 0.391 |  |
| At least one microbleed | 13.48(SD 16.67) | | 14.9(SD 18.23) | | 0.469 |  |
| Lacunar subtype | 13.27(SD 9.26) | | 15.82(SD 22.78) | | 0.487 |  |
| Hypertension | 15.87(SD 20.49) | | 11.63(SD 8.2) | | 0.045 |  |
| Diabetes | 15.87(SD 12.85) | | 14.5(SD 18.53) | | 0.451 |  |
|  | ***With parameter*** | | ***Without parameter*** | | ***P*** |  |
| Stroke | 12.02(SD 7.72) | | 15.02(SD 18.91) | | 0.331 |  |
| PVD | 13.4(SD 8.4) | | 14.74(SD 18.38) | | 0.922 |  |
| eGFR <60 | 22.07(SD 20.82) | | 13.7(SD 17.41) | | ***0.028*** |  |
| Had not cut down on salt | 14.43(SD 20.75) | | 15(SD 13.75) | | 0.613 |  |
|  | ***Spearmans***  ***Rho*** | | ***p*** | | |  |
| Age | 0.21 | | ***<0.01*** | | |  |
| Systolic BP | 0.04 | | 0.58 | | |  |
| Diastolic BP | -0.001 | | 0.93 | | |  |
| NIHSS at worst point | 0.04 | | 0.62 | | |  |
| Total Fazekas score | 0.05 | | 0.45 | | |  |

Table S3: Multivariable analysis of total salt score

|  |  | ***Model 1*** | | ***Model 2*** | ***Model 3*** |
| --- | --- | --- | --- | --- | --- |
| ***Odds ratio of having a lacunae on index scan*** | | | | | |
| Total salt score |  | 1.05(0.94,1.17) | | 1.07(0.95,1.19) | 1.05(0.94,1.18) |
| Age |  |  | | 0.99(0.97,1.02) | 0.99(0.97,1.02) |
| Male gender | |  | | ***2.41(1.32,4.57)*** | ***2.35(1.25,4.56)*** |
| Known hypertension | |  | |  | 1.64(0.82,3.44) |
| Smoker (or recent ex-smoker) | |  | |  | ***2(1.05,3.82)*** |
| AF |  |  | |  | 0.54(0.14,1.6) |
| PVD |  |  | |  | 2.08(0.61,6.73) |
| IHD |  |  | |  | 1.25(0.58,2.64) |
| Hyperlipidaemia | |  | |  | 1.56(0.83,3) |
| ***Odds ratio of WMH*** | | | | | |
| Total salt score |  | 1.09(0.99,1.2) | | 1.07(0.96,1.2) | 1.08(0.96,1.21) |
| Age |  |  | | ***1.09(1.06,1.12)*** | ***1.09(1.06,1.13)*** |
| Male gender | |  | | 0.93(0.53,1.64) | 0.89(0.49,1.6) |
| Known hypertension | |  | |  | ***2.39(1.2,4.89)*** |
| Smoker (or recent ex-smoker) | |  | |  | 1.51(0.8,2.91) |
| AF |  |  | |  | 0.82(0.31,2.21) |
| PVD |  |  | |  | 1.98(0.59,7.87) |
| IHD |  |  | |  | 1.04(0.5,2.16) |
| Hyperlipidaemia | |  | |  | 0.8(0.44,1.45) |
| ***Odds of having EPVL in the basal ganglia***  Odds of having EPVL in the basal ganglia | | | | | |
| Total salt score |  | 1.02(0.92,1.12) | | 1(0.9,1.12) | 1(0.89,1.11) |
| Age |  |  | | ***1.07(1.05,1.1)*** | ***1.08(1.05,1.11)*** |
| Male gender | |  | | 1.68(0.97,2.94) | 1.62(0.92,2.87) |
| Known hypertension | |  | |  | 1.37(0.72,2.62) |
| Smoker (or recent ex-smoker) | |  | |  | 1.68(0.91,3.13) |
| AF |  |  | |  | 0.92(0.36,2.44) |
| PVD |  |  | |  | 0.78(0.24,2.59) |
| IHD |  |  | |  | 1.05(0.51,2.15) |
| Hyperlipidaemia | |  | |  | 0.92(0.52,1.62) |
| ***Odds of having, at least, one microbleed***  Odds of having, at least, one microbleed  Odds of having, at least, one microbleed  Odds of having, at least, one microbleed  Odds of having, at least, one microble  Odds of having, at least, one microbleedOdds of having EPVL in the basal ganglia  Odds of having EPVL in the basal ganglia  Odds of having EPVL in the basal ganglia  Odds of having EPVL in the basal ganglia | | | | | |
| Total salt score |  | 1.04(0.91,1.18) | | 1.03(0.9,1.18) | 1.06(0.92,1.22) |
| Age |  |  | | ***1.04(1.01,1.07)*** | ***1.06(1.02,1.1)*** |
| Male gender | |  | | 1.62(0.81,3.37) | 2.04(0.96,4.55) |
| Known hypertension | |  | |  | 2.22(0.92,6.06) |
| Smoking within last year | |  | |  | 1.59(0.71,3.55) |
| AF |  |  | |  | 0.15(0.01,0.79) |
| PVD |  |  | |  | 1.28(0.3,4.57) |
| IHD |  |  | |  | 0.27(0.08,0.77) |
| Hyperlipidaemia | |  | |  | 1.76(0.82,3.93) |
| ***Odds ratio of having an SVD Score of 2 or more*** | | | | | |
| Total salt score |  | 1.04(0.94,1.15) | | 1.03(0.93,1.15) | 1.02(0.92,1.14) |
| Age |  |  | | ***1.05(1.03,1.08)*** | ***1.06(1.04,1.1)*** |
| Male gender | |  | | 1.63(0.95,2.81) | 1.54(0.88,2.72) |
| Known hypertension | |  | |  | ***2.16(1.13,4.24)*** |
| Smoker (or recent ex-smoker) | |  | |  | ***2.25(1.22,4.26)*** |
| AF |  |  | |  | 0.55(0.21,1.4) |
| PVD |  |  | |  | 1.6(0.5,5.69) |
| IHD |  |  | |  | 1.29(0.64,2.6) |
| Hyperlipidaemia | |  | |  | 1.02(0.57,1.8) |
| ***Linear modelling of predictor of total Fazekas score with total salt score beta, 95% CI*** | | | | | |
| Model 1 Unadjusted | | | b=0.181(95%CI-0.001,3.468 p=0.051) | | |
| Model 2 Age and sex adjusted | | | b=0.156(95%CI-0.054,0.365 p=0.144) | | |
| Model 3 Age, sex & hypertension adjusted | | | b=0.185(95%CI-0.024,0.257 p=0.083) | | |
| ***Bold italics*** indicate p<0.05 | | | | | |

Table S4 Multivariable analysis: of the relationship between urinary salt Fazekas score, and SVD score

| **Odds ratio for a Higher Deep WMH Score (Fazekas)** | |  |  |
| --- | --- | --- | --- |
|  | **Model 1** | **Model 2** | **Model 3** |
| Urine Na:Cr | 0.99(0.97-1.0) | 1.00(0.98-1.01) | 0.99(0.98-1.01) |
| Sex (OR in women) |  | 0.79(0.44-1.40) | 0.67(0.37-1.01) |
| ***Age(per year)*** |  | ***0.91(0.89-0.94)*** | ***0.90(0.87-0.93)*** |
| revious Stroke |  |  | 0.76(0.32-1.77) |
| Hypertension |  |  | 0.80(0.42-1.54) |
| Smoker |  |  | 0.61(0.32-1.14) |
| Family History |  |  | 0.70(0.39-1.25) |
| IHD |  |  | 0.64(0.31-1.29) |
| **Odds ratio for a Higher Periventricular WMH Score (Fazekas)** | |  |  |
|  | **Model 1** | **Model 2** | **Model 3** |
| Urine Na:Cr | 0.98(0.96-1.00) | 0.99(0.97-1.01) | 0.99(0.97-1.00) |
| Sex (OR in women) |  | 0.91(0.51-1.61) | 0.78(0.42-1.43) |
| ***Age(per year)*** |  | 0.99(0.97-1.01) | ***0.91(0.88-0.94)*** |
| ***Previous Stroke*** |  |  | ***0.32(0.13-0.75)*** |
| Hypertension |  |  | 0.80(0.42-1.54) |
| Smoker |  |  | 0.60(0.31-1.103 |
| Family History |  |  | 0.96(0.54-1.71) |
| On diuretics |  |  | 1.45(0.73-2.88) |
| IHD |  |  | 1.10(0.54-2.23) |
| **Odds ratio for a Higher Total WMH Score (Fazekas)** | |  |  |
|  | **Model 1** | **Model 2** | **Model 3** |
| Urine Na:Cr | 0.98(0.97-1.0) | 0.99(0.98-1.01) | 0.99(0.97-1.00) |
| Sex (OR in women) |  | 0.91(0.53-1.56) | 0.79(0.44-124) |
| ***Age(per year)*** |  | ***0.92(0.89-0.94)*** | ***0.90(0.87-0.93)*** |
| Previous Stroke |  |  | ***0.44(0.19-1.00)*** |
| Hypertension |  |  | 0.76(0.40-1.46) |
| Smoker |  |  | 0.64(0.34-1.20) |
| Family History |  |  | 0.70(0.24-1.35) |
| On Diuertics |  |  | 1.85(0.92-3.7) |
| IHD |  |  | 0.67 (0.33-1.37) |
| **Odds ratio of Increased score on SVD Scale** | | | |
|  | **Model 1** | **Model 2** | **Model 3** |
| Na/Cr Ratio | 1.01(0.83-1.23) | 0.88(0.7-1.08) | 0.99(0.80-1.23) |
| ***Age(per year)*** |  | 1.06(1.04-1.10) | 1.06(1.03-1.09) |
| On Diueretics |  |  | 0.61(0.30-1.20 |
| MAP |  |  | 1.01(0.99-1.02) |
